# A simple chest CT score for assessing the severity of pulmonary involvement in COVID-19

**DOI:** 10.1186/s43055-021-00525-x

**Published:** 2021-06-18

**Authors:** Mohamed Abdel-Tawab, Mohammad Abd Alkhalik Basha, Ibrahim A. I. Mohamed, Hamdy M. Ibrahim

**Affiliations:** 1 Department of Diagnostic Radiology, Assiut University, Assiut, 71515 Egypt; 2 Department of Radio-diagnosis, Faculty of Human Medicine, Zagazig University, Zagazig, Egypt

**Keywords:** Protein corona, Pneumonia, viral, Tomography, X-ray computed, Validation study, Reproducibility of results

## Abstract

**Background:**

A major role of CT in COVID-19 pneumonia is to assess disease severity and progress. In this study, we aimed to assess the validity, reliability, and survival outcomes of simple chest computed tomography (CT) score in the evaluation of the severity of lung involvement in coronavirus disease 2019 (COVID-19) compared with the current chest CT score.

**Results:**

This retrospective analysis included 213 patients (121 men and 92 women; mean age, 46 ± 15.6 years; range, 1–85 years). The ROC curve was used to compare the validity of both scores. Interreader agreement (IRA) for both scores was calculated using Cohen’s kappa statistic. The survival analysis of both scores was investigated using the Kaplan–Meier survival analysis. The simple score showed a comparable validity with the current score (AUC = 0.89 and 0.90, respectively; *p* = 0.61). The ROC analysis demonstrated that a simple score of > 3 and a current score of > 12 were potential predictors of death with sensitivity values of 81.8% and 86.4% and specificity values of 96.3% and 93.7%, respectively. The simple score showed a higher IRA compared with the current score (κ = 0.645 and 0.458, respectively). Both scores were comparable for predicting survival outcomes.

**Conclusion:**

The simple score was non-inferior for predicting survival outcome, compared with the current chest CT score. Furthermore, we suggest that the simple score should be used as it is simpler and more consistent.

## Background

Since the outbreak and the global spread of acute respiratory syndrome in Wuhan, China, in late December 2019 [1], the World Health Organization has announced coronavirus disease 2019 (COVID-19) as a pandemic in March 2020 [2]. COVID-19 continues spreading all over the world, reaching 215 countries with greater than 115 million cases and 2.5 million deaths as of March 2, 2021 [3], which represents a significant challenge to international health agencies [4]. As of now, the world is facing a second-wave outbreak of COVID-19 [5] that urges us to refine our management plans.

The severity of COVID-19 in patients at risk should be classified to prioritize medical resources in hospitals, especially when resources and medical staff are limited [6]. Several studies have developed severity scoring systems that evaluate the percentage of pulmonary involvement in COVID-19 [7–10]. The computed tomography (CT) severity score (CT-SS) was developed by Yang et al. [7]. The score is an adaptation of a method previously used during the severe acute respiratory syndrome epidemic in 2005 [11]. The 18 segments of both lungs were divided into 20 regions in this score. Then, the lung opacities were subjectively assessed on chest CT in all of the 20 lung regions. Each region was given 0, 1, or 2 points based on the parenchymal opacification involved: 0%, 1–50%, or 51–100%, respectively. The overall CT-SS was described as the sum of the points obtained in each of the 20 lung regions, which ranges from 0 to 40 points. The total severity score (TSS) was developed by Kunwei Li et al. [8]. Each lobe could be assigned 0 to 4 points in this score based on the proportion of the involved lobe: 0 (0%), 1 (1–25%), 2 (26–50%), 3 (51–75%), or 4 (76–100%). The TSS was then attained by summing the points from each of the five lobes. Another severity scoring system is the chest CT score, which was proposed by Kunhua Li et al. [9] and it is the most widely used score by many radiologists. Similarly to the TSS, in this score, each lobe could be awarded a CT score from 0 to 5, depending on the percentage of the involved lobe: score 0, 0% involvement; score 1, < 5% involvement; score 2, 5–25% involvement; score 3, 26–49% involvement; score 4, 50–75% involvement; and score 5, > 75% involvement. The total CT score was the sum of the points from each lobe and ranges from 0 to 25 points. The semi-quantitative CT-SS was proposed by Pan et al. [10]; in this score, similar to the other severity scores, each lung lobe was visually rated on a scale of 0 to 5, with 0 denoting no involvement and 5 denoting > 75% involvement. The overall CT score was the sum of the individual lobar scores and varied from 0 (no involvement) to 25 (maximum involvement).

Although the application of these scores was a significant advance in the evaluation of the severity of COVID-19, these scores have some limitations. First, they are complicated and hence time-consuming. The wide scoring range from 20 to 40 regions in some scores increases the difficulty of the evaluation. Second, the sizes between both lungs are different, with the right larger than the left, including their corresponding lobes and segments themselves. Even the quantitative methods that calculate the volume of pneumonic lesions need dedicated software and an experienced operator [12]. The aim of this study, therefore, is to simplify the chest CT score and evaluate the validity, reliability, and survival outcomes of the proposed simple chest CT score in comparison with the most widely used scoring system which is the current chest CT score.

## Methods

### Patient population

This retrospective, single-center study included 213 COVID-19-positive patients confirmed by reverse transcription-polymerase chain reaction of nasal and throat swab specimens. The institutional review board approved this study and waived the need to gather patients’ formal consent. The patients were referred and admitted between May 2020 and June 2020. In this study, the patients with swab-confirmed COVID-19 who underwent chest CT imaging within 12 h after admission were included. We excluded patients with CT imaging performed before hospital admission or those with poor image quality.

### CT imaging

All scans were performed using a 16-channel CT scanner (Aquilion Lightning; Toshiba Medical Systems) with the following scanning parameters: tube voltage 120 kV; tube current 50 mA; rotation time 0.5 s; slice thickness 5 mm; matrix 512 × 512. Each patient was scanned in the supine position during breath-hold on full inspiration without intravenous contrast. The scanning range was from the apex to the lung base.

### CT image analysis

Two expert chest radiologists (10 years of experience in chest radiology) with no knowledge of the signs and symptoms or the patient outcomes, retrospectively and independently evaluated all CT scans on the PACS workstation (Vitrea® Advanced Visualization; Vital Images). The initial chest CT was evaluated for each patient for the following features: lesion distribution, interlobular septal thickening, ground-glass opacities (GGO), consolidation, crazy-paving pattern, and reverse halo sign. The severity of the pulmonary involvement for each patient was evaluated by each radiologist using the chest CT score established by Kunhua Li et al. [9]. Subsequently, the severity of the pulmonary involvement was assessed by each radiologist using a simple chest CT score, which is similar to the TSS and the current chest CT score but differs with the addition of a simplified assessment of the degrees of involvement of both lungs as a whole. Using this simple score, the degree of lung involvement for each patient was then classified as score 0 (0% or none), score 1 (1–5% or minimal), score 2 (6–25% or mild), score 3 (26–49% or moderate), score 4 (50–74% or severe), and score 5 (≥ 75% or extensive). Radiologists assigned the simple score after reviewing both lungs as a whole in axial, coronal, and sagittal reconstruction planes to accurately quantify the amount of affection.

### Statistical analysis

Statistical Package for the Social Sciences version 26 and MedCalc Software version 18.2.1 were used for statistical analysis. Categorical variables were represented as numbers and percentages, and statistical significance was calculated using Fisher’s exact test. Continuous data were expressed in mean ± standard deviation. ROC curve analysis was performed to determine the cutoff values and calculate the validity of both scores in predicting mortality. The interreader agreement (IRA) was evaluated using Cohen’s kappa statistic, and k values were defined as follows: 0–0.2 = no agreement; 0.21–0.4 = weak agreement; 0.41–0.60 = moderate agreement; 0.61–0.80 = good agreement; and 0.81–1.0 = excellent agreement. A Kaplan–Meier survival analysis with a log-rank test was conducted to assess the time to death. Statistical significance was described as *p*-value < 0.05.

## Results

### Patients

The analysis included 213 patients (121 men and 92 women; mean age, 46 ± 15.6 years; range, 1–85 years). After a short-term follow-up (mean, 13.8 ± 3.9 days; range, 3–20 days), 22 (10.3%) patients died, and 191 (89.7%) patients showed improvement and were discharged. The description of the demographic and clinical features of the study population is summarized in Table 1.

**Table 1:** Demographic and clinical characteristics of study population

Characteristic	All (*n* = 213)	Death (*n* = 22)	Survival (*n* = 191)	*P*-value
Age, years, mean ± SD (range)	45 ± 16.8 (1–85)	59.3 ± 14.0 (30–85)	43.4 ± 16.2 (1–80)	< 0.001
Gender				
Male	121 (56.8)	14 (63.6)	107 (56.0)	0.509
Female	92 (43.2)	8 (36.4)	84 (44.0)	
Symptoms				
Fever	173 (81.1)	22 (100)	151 (79.1)	0.017
Cough	155 (72.8)	20 (90.9)	135 (70.7)	0.045
Dyspnea	102 (47.9)	19 (86.4)	81 (42.4)	< 0.001

Unless otherwise indicated, data represent number and percentage in parenthesis

*ST* standard deviation

### CT image findings

**Fig. 1:**
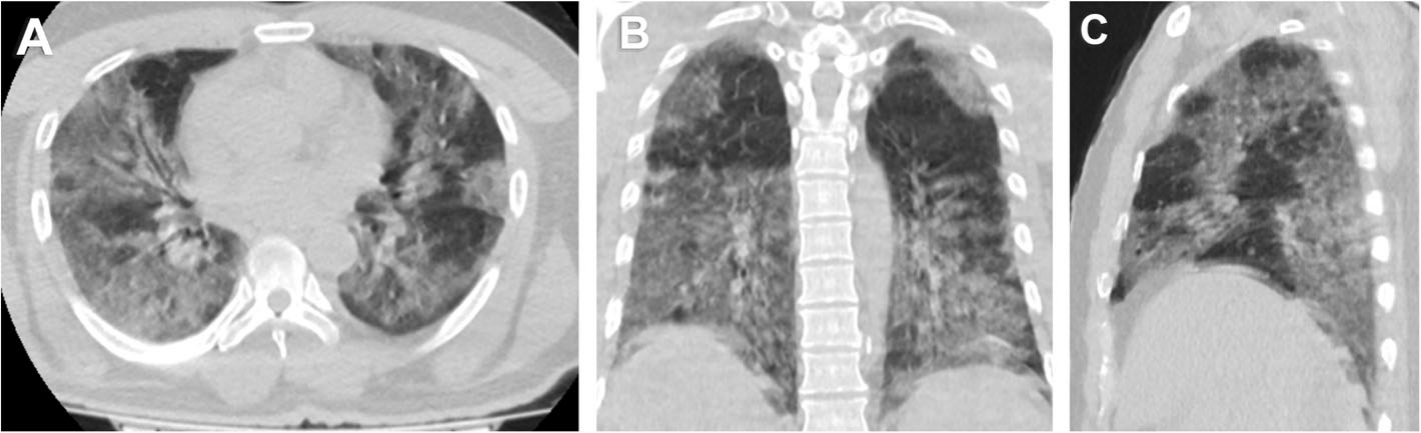
A 52-year-old male patient. **A** axial, **B** coronal, and **C** sagittal chest CT images show bilateral diffuse ground-glass opacities and consolidation with extensive pulmonary involvement (> 75%) corresponds to current chest CT score 20 and simple chest CT score 5 (extensive affection). The patient died on the sixth day

**Fig. 2:**
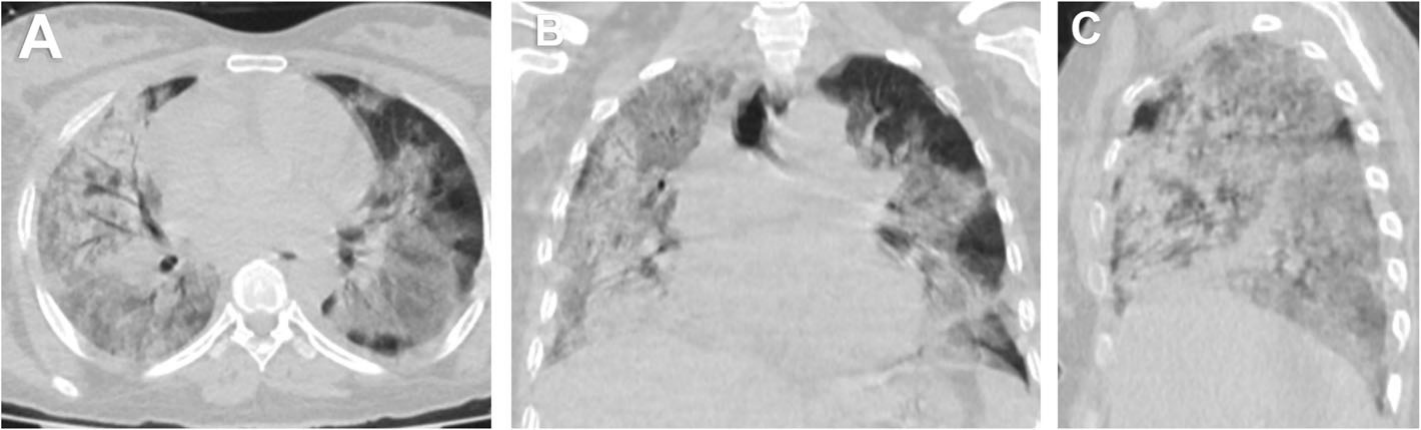
A 30-year-old female patient. **A** axial, **B** coronal, and **C** sagittal chest CT images show bilateral diffuse ground-glass opacities and consolidation with extensive pulmonary involvement (> 75%) corresponds to current chest CT score 22 and simple chest CT score 5 (extensive affection). The patient died on the fifth day

**Fig. 3:**
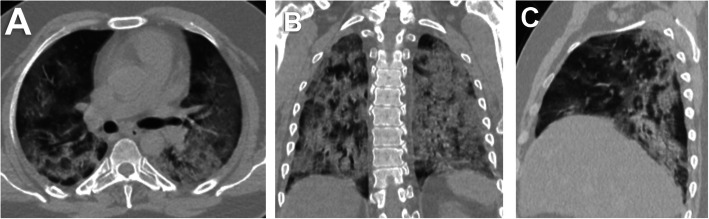
A 44-year-old male patient. **A** axial, **B** coronal, and **C** sagittal chest CT images show bilateral diffuse mainly peripheral ground-glass opacities and consolidation with severe pulmonary involvement (50-75%) corresponds to current chest CT score 19 and simple chest CT score 4 (severe affection) and. The patient survived

**Fig. 4:**
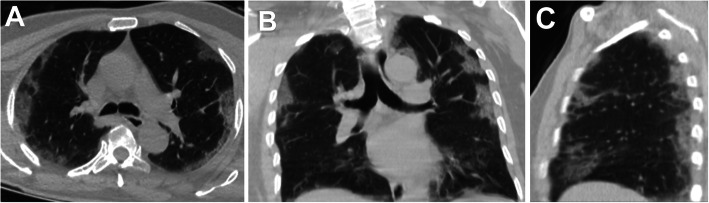
A 60-year-old female patient. **A** axial, **B** coronal, and **C** sagittal chest CT images show bilateral ground-glass opacities and consolidation with moderate pulmonary involvement (26–49%) corresponds to current chest CT score 13 and simple chest CT score 3 (moderate affection). The patient died on the seventeenth day

**Fig. 5:**
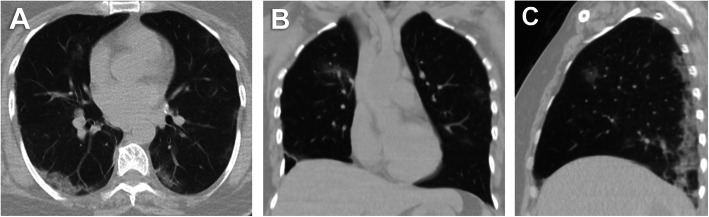
A 50-year-old female patient. **A** axial, **B** coronal, and **C** sagittal chest CT images show bilateral peripheral ground-glass opacities and consolidation with mild pulmonary involvement (5–25%) corresponds to current chest CT score 8 and simple chest CT score 2 (mild affection). The patient survived

**Fig. 6:**
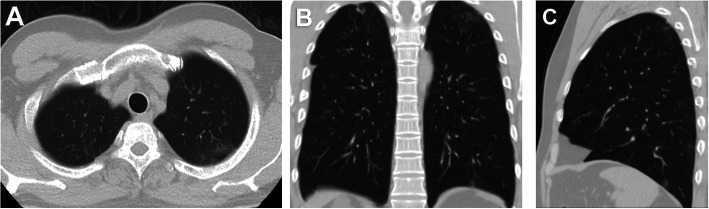
A 53-year-old female patient. **A** axial, **B** coronal, and **C** sagittal chest CT images show bilateral peripheral small ground-glass opacities with minimal pulmonary involvement (< 5%) corresponds to current chest CT score 4 and a simple chest CT score 1 (minimal affection). The patient survived

Fifty-two (24.4%) patients had no pulmonary changes in the CT images, whereas 161 (75.6%) patients had pneumonic changes. The most frequent image finding was GGO (71.8%), followed by consolidation (35.7%). The distribution of the lesions was peripheral in 51.6% of the patients and diffuse in 23.9% of the patients. A crazy-paving pattern was observed in 7% of the patients and a reverse halo in 1.9% of the patients. No patient had pleural effusion. The incidence rates of diffuse distribution, multifocal affection, GGO, consolidation, and crazy-paving pattern were higher in the death group. The reverse halo sign was observed in the survival group only (2.1%) (Table 2) (Figs. 1, 2, 3, 4, 5, and 6).

**Table 2:** CT image findings

Findings	Death group (*n* = 22)	Survival group (*n* = 191)	*P*-value
Distribution			< 0.001
Peripheral	4 (18.2)	106 (55.5)	
Diffuse	18 (81.8)	31 (16.2)	
GGO	22 (100)	131 (65.4)	< 0.001
Multifocal	22 (100)	125 (100)	< 0.001
Consolidation	17 (77.3)	59 (30.9)	< 0.001
Septal thickening	6 (27.3)	45 (23.6)	0.699
Crazy paving	4 (18.2)	11 (5.8)	< 0.001
Reverse halo	0 (0)	4 (2.1)	1.000

Data represent the number of patients and percentage in parenthesis

*CT* computed tomography, *GGO* ground-glass opacity

### Assignment of both scores stratified by survival outcome

According to both scores stratified by patient survival outcome. Both scores were significantly higher in the death group (*p* = 0.000) (Table 3).

**Table 3:** Assignment of both scores stratified by survival outcome

Score	Total (*n* = 213)	Death group (*n* = 22)	Survival group (*n* = 191)	*P*-value
Current CT score				< 0.001
0	55 (25.8)	0 (0)	55 (28.8)	
1-5	51 (23.9)	1 (4.5)	50 (26.2)	
6-10	54 (25.4)	1 (4.5)	53 (27.7)	
11-15	31 (14.6)	4 (18.2)	27 (14.1)	
16-20	17 (7.9)	13 (59.1)	4 (2.1)	
> 20	5 (2.3)	3 (13.6)	2 (1)	
Simple CT score				< 0.001
0	55 (25.8)	0 (0)	55 (28.8)	
1	47 (22.1)	1 (4.5)	46 (24.1)	
2	55 (25.8)	1 (4.5)	54 (28.3)	
3	31 (14.6)	2 (9.1)	29 (15.2)	
4	18 (8.5)	13 (59.1)	5 (2.6)	
5	7 (3.3)	5 (22.7)	2 (1)	

Data represent the number of patients and percentage in parenthesis

*CT* computed tomography

### Validity of both scores

**Fig. 7:**
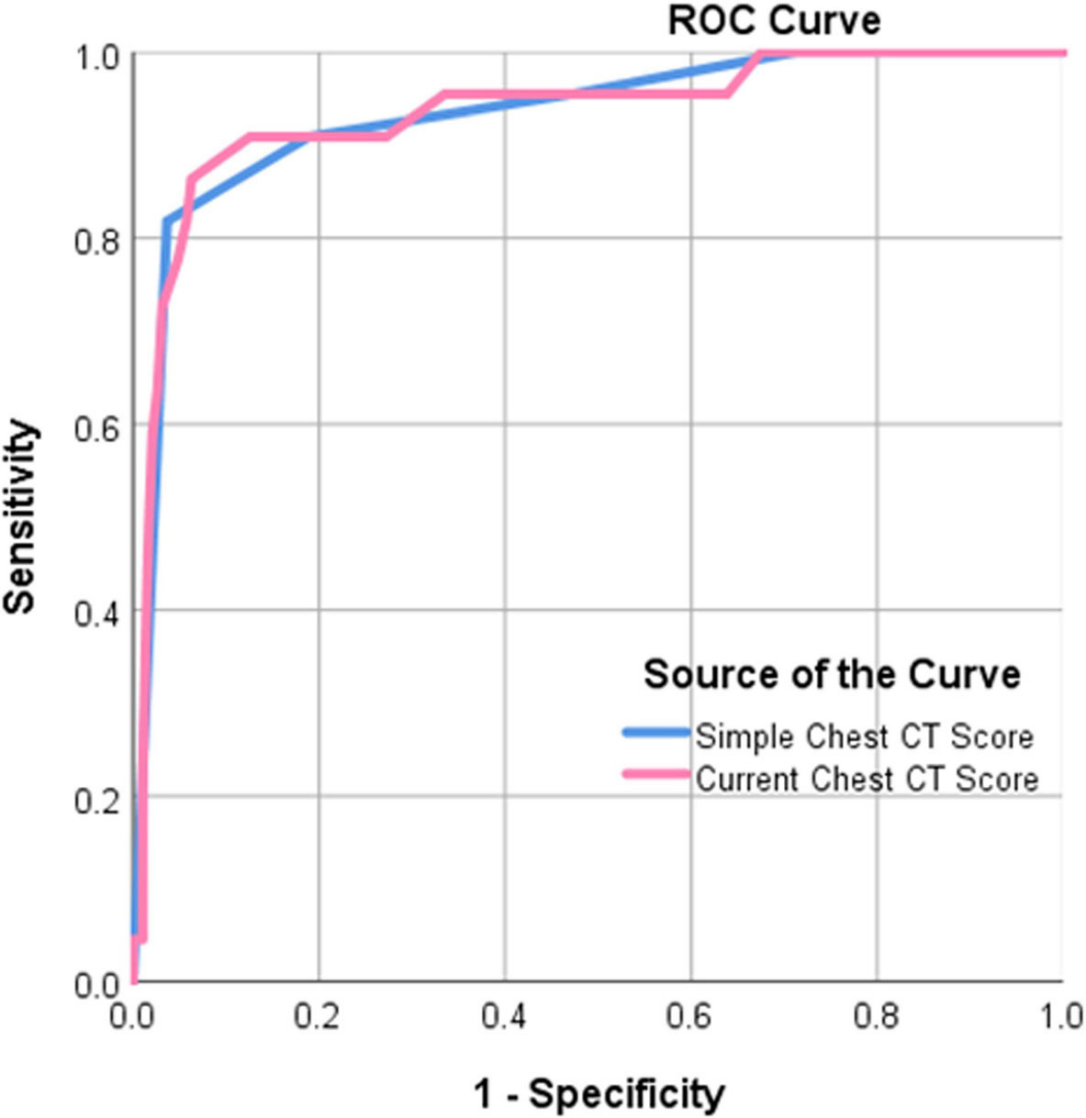
Receiver operator characteristic (ROC) curve for chest CT scores in predicting mortality

The analysis of the validity of both scores to determine the optimal cutoff value for predicting survival outcomes using the ROC curve is shown in Fig. 7. The areas under the curve were 0.89 and 0.90 with the optimal cutoff value for predicting survival outcomes being > 3 and >  12 for the simple and current scores, respectively. Table 4 summarizes the validity of both scores for predicting survival outcomes using the consensus data. A simple score of > 3 and a current score of > 12 were potential predictors of death with sensitivity values of 81.8% and 86.4% and specificity values of 96.3% and 93.7%, respectively (*p* > 0.05).

**Table 4:** Validity of both scores for predicting survival outcome using the consensus data

	Simple CT score	Current CT score	*P*-Value
Cutoff	> 3	> 12	
Number of true-positive findings	18	19	
Number of false-negative findings	4	3	
Number of false-positive findings	7	12	
Number of true- negative findings	184	179	
AUC	0.89 [0.84–0.93]	0.90 [0.85–0.94]	0.615
Sensitivity (%)	81.8 (18/22) [59.7–94.8]	86.4 (19/22) [65.1–97.1]	0.244
Specificity (%)	96.3 (184/191) [92.6–98.5]	93.7 (179/191) [89.3–96.7]	0.313
PPV (%)	72 (18/25) [50.6–87.9]	61.3 (19/31) [47.2–73.7]	0.025
NPV (%)	97.9 (184/188) [94.6–99.4]	98.4 (179/182) [95.3–99.6]	0.981
Accuracy (%)	94.8 (202/213) [91.5–98.4]	93 (198/213) [89.2–96.1]	0.566

Data in parentheses were used to calculate percentages. Data in brackets are 95% confidence intervals

*CT* computed tomography, *PPV* positive predictive value, *NPV* negative predictive value, *AUC* area under curve

### Reliability of both scores

The simple score was more consistent between the readers than the current score. The IRA was good for the simple score (κ = 0.645, 95% CI = 0.643–0.647) and moderate for the current score (κ = 0.458, 95% CI = 0.457–0.459).

### Survival analysis

**Fig. 8:**
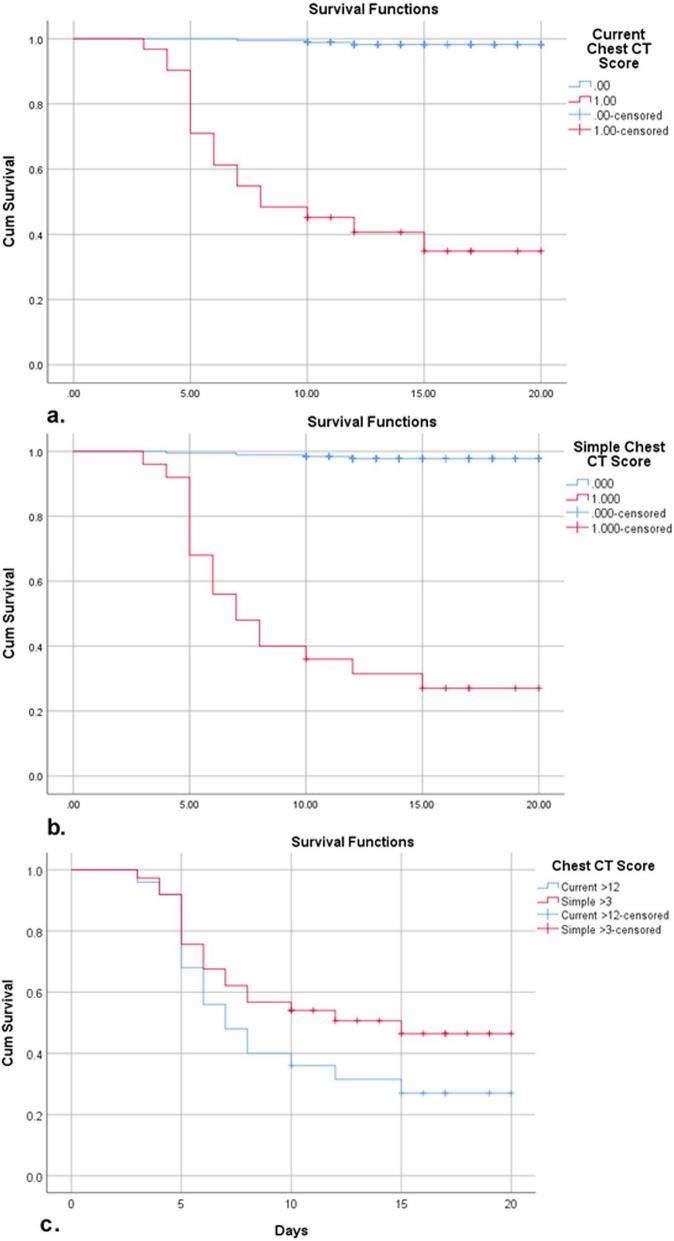
The Kaplan–Meier survival curves show the comparison of survival rates. **a** Comparison of estimated survival rate between patients with a score of ≤12 and patients with a score of > 12 using current chest CT score. **b** Comparison of estimated survival rate between patients with a score of ≤3 and patients with a score of > 3 using simple chest CT score. **c** Comparison between two scores regarding estimated survival rate

The Kaplan–Meier survival curves (Fig. 8) indicated that the survival rates in the patients with a current score of > 12 and a simple score of > 3 were significantly lower than those in patients with a current score of ≤12 and a simple score of ≤3, respectively (*p* <  0.0001). However, the log-rank test revealed that both scores were comparable for predicting survival outcomes with no statistically significant difference (*p* = 0.146).

## Discussion

With the rapid spread of the COVID-19 pandemic, the development of a standardized score with fixed criteria is essential to improve the consistency of the interpretations of chest CT images among radiologists [13, 14]. Regarding the complexity and time-consuming application of the current scores in clinical practice, we hypothesized a simple score that is a simplification of a well-known current chest CT score. This simple score would be useful and convenient for evaluating the severity of lung involvement in COVID-19. A comparison of the criterion-fixed score systems and text-free scales is needed. In this study, we compared the simple and current chest CT scores concerning their validity, reliability, and survival outcomes for evaluating the severity of lung involvement in COVID-19. Moreover, our study showed that the simple score that takes only a few minutes to administer is a valid and reliable score for evaluating the severity of lung involvement in COVID-19.

Our study compared the validity of the two scores in detecting the severity of lung involvement in COVID-19 and found that no statistically significant differences were observed between the two scores. Both scores had shown great value in the evaluation of the severity of lung involvement in COVID-19 with statistically comparable sensitivity and specificity. We found that a simple score cutoff value of > 3 predicted death with a sensitivity of 81.8% and specificity of 96.3% and a current score cutoff value of > 12 predicted death with a sensitivity of 86.4% and specificity of 93.7%. Our results were comparable with those of previous studies [7–10, 15] about the correlation of the current scores with the clinical severity or patient survival. Yang et al. [7] using the CT-SS revealed a sensitivity of 83.3% and specificity of 94% with a cutoff value of 19.5. Kunwei Li et al. [8] using the TSS revealed a sensitivity of 82.6% and specificity of 100% with a cutoff value of 7.5. Kunhua Li et al. [9] using the current chest CT score revealed a sensitivity of 80% and specificity of 82.8% with a cutoff value of 7. Francone et al. [14] using a semi-quantitative CT-SS proposed by Pan et al. [10] found that a CT-SS of ≥18 was associated with increased mortality risk.

Interreader reliability is essential for assessing any new scoring system. The results of this study showed higher IRA in the evaluation of the severity of lung involvement in COVID-19 when using the simple score compared with the current score. The IRA between two experienced readers for the simple score was good (k = 0.645) and for the current score was moderate (k = 0.458). This better reliability of the simple score compared with the current score could be attributed to the inherent simplifications of this score. Moreover, the more regions assessed in the current score may increase the variability. Additionally, good IRA may be due to the higher experience of readers in our study. However, this is potentially affecting the validity of the simple score. Thus, further studies on the validity of this score when applied by less experienced radiologists are needed. Previous studies [7, 8] reported excellent IRA for the current score. Yang et al. [7] reported excellent IRA for the CT-SS with an intraclass correlation coefficient (ICC) of 0.925. Kunwei Li et al. [9] reported good repeatability for the TSS with an ICC of 0.976.

Our survival curve analysis demonstrated that both scores had comparable high values in predicting survival outcomes in patients with COVID-19 with no statistically significant difference (*p* = 0.146). Similar findings were reported by previous studies [15, 16], which found a positive correlation between the extent of CT lung involvement and short-term mortality.

In the present study, we found that the most common CT findings were the peripheral distribution, multifocal affection, GGO, consolidation, and crazy-paving pattern, which were more frequent in the death group. These findings are similar to that of previous studies [9, 16]. Death in patients with mild scores may be attributed to causes other than pneumonia, e.g., pulmonary embolism that will not be depicted in non-contrast CT [17].

Our study had some notable limitations. First, it was a single-center experience with a retrospective design, so patient selection biases could have been present. Second, from the consecutive patient cohort diagnosed with COVID-19 in our institution, we included only the subgroup of patients who underwent CT within 12 h after admission. Hence, to confirm the true validity of the simple chest CT score, the score probably has to be assessed prospectively in all patients with COVID-19 at the time of presentation. However, because not all patients with COVID-19 need to have a CT examination, the methodology used in our study indicates, in some respects, current clinical practice. Third, all CT images were obtained within 12 h after admission regardless of the onset of symptoms. Fourth, we did not consider the impact of comorbidity factors on CT severity. Finally, no long-term follow-up data were available.

## Conclusions

Overall, no statistically significant difference was observed between the validity of the two scores. Nevertheless, we suggest that the simple chest CT score be used as it is simpler and more consistent. We recommend using the corresponding descriptive term (i.e., minimal, mild, moderate, severe, and extensive) beside the score to clarify the degree of affection.

## Data Availability

The data that support the findings of this study are available from Radiology Department—Assiut University, but there are restrictions that apply to the availability of data, which are used under license for this study, and so were not publicly available. Data were available from authors upon request with permission of the head of the Radiology Department—Assiut University.

## Abbreviations

AUC: Area under the curve
CT: Computed tomography
CT-SS: Computed tomography severity score
COVID-19: Coronavirus disease 2019
GGO: Ground-glass opacities
IRA: Interreader agreement
NPV: Negative predictive value
PPV: Positive predictive value
ROC: Receiver operating characteristic
TSS: Total severity score

## References

[1] Wu F, Zhao S, Yu B (2020). A new coronavirus associated with human respiratory disease in China. Nature.

[2] WHO announces COVID-19 outbreak a pandemic. https://www.euro.who.int/en/health-topics/health-emergencies/coronavirus-covid-19/news/news/2020/3/who-announces-covid-19-outbreak-a-pandemic. Accessed 31 Aug 2020

[3] (2020) Coronavirus toll update: cases and deaths by country and territory. https://www.worldometers.info/coronavirus/. Accessed 3 Nov 2020

[4] Kandel N, Chungong S, Omaar A, Xing J (2020). Health security capacities in the context of COVID-19 outbreak: an analysis of international health regulations annual report data from 182 countries. Lancet.

[5] Middleton J, Lopes H, Michelson K, Reid J (2020) Planning for a second wave pandemic of COVID-19 and planning for winter: a statement from the Association of Schools of Public Health in the European Region. Int J Public Health 10.1007/s00038-020-01455-7 PMC7453079 32857238

[6] Fu F, Lou J, Xi D (2020). Chest computed tomography findings of coronavirus disease 2019 (COVID-19) pneumonia. Eur Radiol.

[7] Yang R, Li X, Liu H (2020). Chest CT severity score: an imaging tool for assessing severe COVID-19. Radiol Cardiothorac Imaging.

[8] Li K, Fang Y, Li W (2020). CT image visual quantitative evaluation and clinical classification of coronavirus disease (COVID-19). Eur Radiol.

[9] Kunhua Li WJ, Wu F (2020). The clinical and chest CT features associated with severe and critical COVID-19 pneumonia. Investig Radiol.

[10] Pan F, Ye T, Sun P (2020). Time course of lung changes at chest CT during recovery from coronavirus disease 2019 (COVID-19). Radiology.

[11] Chang YC, Yu CJ, Chang SC (2005). Pulmonary sequelae in convalescent patients after severe acute respiratory syndrome: evaluation with thin-section CT. Radiology.

[12] Yin X, Min X, Nan Y (2020). Assessment of the severity of coronavirus disease: quantitative computed tomography parameters versus semiquantitative visual score. Korean J Radiol.

[13] Abdel-Tawab M, Basha MAA, Mohamed IAI (2021). Comparison of the CO-RADS and the RSNA chest CT classification system concerning sensitivity and reliability for the diagnosis of COVID-19 pneumonia. Insights Imaging.

[14] Mohamed IAI, Hasan HA, Abdel-Tawab M (2021). CT characteristics and laboratory findings of COVID-19 pneumonia in relation to patient outcome. Egypt J Radiol Nucl Med.

[15] Francone M, Iafrate F, Masci GM et al (2020) Chest CT score in COVID-19 patients: correlation with disease severity and short-term prognosis. Eur Radiol. 10.1007/s00330-020-07033-y 10.1007/s00330-020-07033-y PMC7334627 32623505

[16] Colombi D, Bodini FC, Petrini M (2020). Well-aerated lung on admitting chest ct to predict adverse outcome in COVID-19 pneumonia. Radiology.

[17] Gervaise A, Bouzad C, Peroux E, Helissey C (2020) Acute pulmonary embolism in non-hospitalized COVID-19 patients referred to CTPA by emergency department. Eur Radiol. 10.1007/s00330-020-06977-5 10.1007/s00330-020-06977-5 PMC7280685 32518989

